# Injectable polypeptide hydrogel/inorganic nanoparticle composites for bone tissue engineering

**DOI:** 10.1371/journal.pone.0210285

**Published:** 2019-01-10

**Authors:** Wei-Shun Huang, I-Ming Chu

**Affiliations:** Department of Chemical Engineering, National Tsing Hua University, Hsinchu, Taiwan; Brandeis University, UNITED STATES

## Abstract

The general concept of tissue engineering is to restore biological function by replacing defective tissues with implantable, biocompatible, and easily handleable cell-laden scaffolds. In this study, osteoinductive and osteoconductive super paramagnetic Fe_3_O_4_ nanoparticles (MNP) and hydroxyapatite (HAP) nanoparticles were incorporated into a di-block copolymer based thermo-responsive hydrogel, methoxy(polyethylene glycol)-polyalanine (mPA), at various concentrations to afford composite, injectable hydrogels. Incorporating nanoparticles into the thermo-responsive hydrogel increased the complex viscosity and decreased the gelation temperature of the starting hydrogel. Functionally, the integration of inorganic nanoparticles modulated bio-markers of bone differentiation and enhanced bone mineralization. Moreover, this study adopted the emerging method of using either a supplementary static magnetic field (SMF) or a moving magnetic field to elicit biological response. These results demonstrate that combining external (magnet) and internal (scaffold) magnetisms is a promising approach for bone regeneration.

## Introduction

Trauma, bone disease, and tumor excision are common causes of critical-sized bone defects. Currently, few synthetic bone grafts offer osteoconduction and osteoinduction properties comparable to those of bone autographs, which have limited availability [1, 2]. Therefore, bone tissue engineering is a field of significant research interest. The general approach of bone tissue engineering is to offer a biomimetic environment that supports cell adhesion, growth, and proliferation; as well as provide proper physical (e.g., static magnetic field), chemical, and biological (e.g., osteogenic genes, proteins, or cells) factors to support tissue regeneration [3, 4].

Hydrogel is a soft matter with high water content which mimics the extracellular matrix (ECM) found in the body [5]. Specific hydrogels are injectable and can be used to easily fill in defects of various sizes and shapes while retaining their unique physical and biological properties [6, 7]. Peptide hybrid polymers are a promising new class of biomaterials developed to combine the advantages of synthetic and peptide polymers, namely the ability of peptide polymers to self-assemble into hierarchical structures and the simplicity and reproducibility in the preparation of synthetic polymers [8–10]. However, in addition to high water content and permeability for nutrients and oxygen, hydrogels used for bone tissue engineering must also exhibit supportive properties for cell growth and differentiation. Composites that consist of nanomaterials incorporated into a hydrogel are attractive for bone tissue engineering because this strategy allows fine control of the mechanical, biological, and chemical properties of the scaffold [11–13].

Among the nanomaterials, HAP and MNP are commonly employed in bone tissue engineering because these materials have been shown to improve bone tissue mineralization when incorporated into various scaffold materials [14–17]. In addition, they have been adopted specifically as bioactive factors in hydrogels to improve mineralization and osteogenic differentiation [18–22]. Furthermore, MNP have been incorporated into different matrices to fabricate magnetic composites [23–26]. In fact, many research groups have reported that weak magnetic or pulse electromagnetic fields induce and promote bone fracture healing, spinal fusion, and bone ingrowth into ceramics in animal models. Moreover, a strong static magnetic field of 0.1–10 T has been shown to regulate the orientation of matrix proteins and cells *in vitro* and *in vivo* [27–29].

In this study, we introduced both HAP and MNP into a mPEG-poly(alanine) hydrogel to provide bone tissue engineering scaffolds of suitable mechanical property and compatibility. Then, magnetic stimulation was applied to explore the potential positive effects of this strategy in combination with the composite approach. The performance was evaluated based on gene expression and cell staining.

## Materials and methods

### Materials

Methoxy polyethylene glycol of Mw 2000 (mPEG), l-alanine, dimethyl sulfoxide (DMSO), potassium hexacyanoferrate(II) trihydrate, trypsin-EDTA solution 0.25%, dexamethasone, β-glycerophosphate, ascorbic acid, iron (II) sulfate heptahydrate, iron (III) chloride, calcium chloride, disodium hydrogen phosphate, phosphate buffer saline (PBS), tetramethylammonium hydroxide (TMAH) solution (25 wt%), LIVE/DEAD assay kit and deuterated trifluoracetic acid (TFA-d) were obtained from Sigma (St. Louis, MO). N,N-dimethylformamide (DMF, Sigma- Aldrich), tetrahydrofuran (THF, Sigma-Aldrich), and chloroform (Mallinckrodt) were dried over CaH2 before use. SYBR Green Real-time PCR Master Mix was received from Applied Biosystems (Carlbad, CA). Primers were designed in lab and synthesized by MDBio (Taipei, Taiwan). Alpha-minimum essential medium (α-MEM), fetal bovine serum (FBS), antimycotics-antibiotics, gentamycin, and fungizone were purchased from Gibco.

### Synthesis of di-block copolymer mPA

Methoxy polyethylene glycol-poly (alanine) (mPA) was synthesized by ring-opening polymerization included amine-terminated mPEG and *N*-carboxyanhydride form of L-alanine. Protocols used to prepare mPEG-NH_2_ and alanine-NCA (Ala-NCA) are similar to those described in a previous study [30, 31]. Briefly, 2.00 g (1mmol) of mPEG was dissolved in anhydrous chloroform/DMF (100 mL; 2/1 v/v) and 4.86g (42.25 mmol) of Ala-NCA was added to the reaction mixture. Polymerization was carried out at 25°C for 72 h under anhydrous nitrogen condition. The theoretical molecule weight of synthesized mPA is 2000–3000. Finally, the product was precipitated, dissolved in DMSO for dialysis against deionized water (MW cut of 3500), and lyophilized.

### Synthesis of magnetic nanoparticles (MNP)

Fe_3_O_4_ MNP were synthesized by chemical co-precipitation, according to a described method, with minor modification [32]. In the typical experimental procedure, MNP were fabricated by mixing 10 mL of 1M ferric chloride hexahydrate (FeCl_3_•6H_2_O) with 5 mL of 1M ferrous sulfate heptahydrate (FeSO_4_•7H_2_O) in a two- neck flask under nitrogen atmosphere. The solution was stirred, followed by the dropwise addition of 20 ml of 25% (w/w) TMAH, until a pH of 13 was reached. Vigorous stirring was continued for 20 min. The solution color changed from orange to black, as black precipitates formed. A permanent magnet was used to isolate the black precipitated product and the supernatant was discarded. Deionized water was added for washing to remove excess ions and tetramethyl ammonium salt in the suspension. This washing procedure was repeated for three times. Finally, the washed precipitated product was dispersed in deionized water and stored until further use.

### Synthesis of hydroxyapatite nanoparticles

Hydroxyapatite (HAP) nanoparticles were synthesized using a method described previously [33]. Briefly, 0.03M Na2HPO4 was added dropwise into 0.05M CaCl2 at 50°C and pH 10 with a stirring speed of 300 rpm. After continuous stirring at 300 rpm for 24 h, the precipitated product was harvested by centrifugation, washed, and dried. The final product was dispersed in deionized water and stored until further investigation.

### Preparation of MNP/mPA and HAP/mPA nanocomposite hydrogels

First, mPA was dissolved in deionized water at 4°C for 24 h to yield a 3 wt% solution. Next, MNP and HAP suspensions were dispersed by a ultrasonicator (DC400, Delta, Taiwan) and separately added into hydrogel solutions at different nanoparticle concentrations (0, 500, and 1000 μg/mL). Then, the mixtures were added dropwisely into cell culture plates and cultured for 10 min in a 37°C oven to afford nanocomposite hydrogels. Nanocomposite hydrogels containing 500 μg/mL of HAP and 1000 μg/mL of MNP are denoted as H5/mPA and M10/mPA, respectively.

### ^1^H-NMR spectroscopy

Copolymer mPA was dissolved in TFA-d prior to analysis. The composition and number average molecular weight (Mn) were verified using a 500 MHz NMR spectrometer (Varian, USA).

### Solution-gel (sol-gel) phase transition

The storage modulus of mPA, M10/mPA and H5/mPA in aqueous solutions (3.0 wt%) was investigated by a rheometer (MCR 302, Anton Paar GmbH, Austria) at 4°C and 37°C. Briefly, aqueous polymer solution was pipetted between parallel plates 25 mm in diameter with a gap of 0.5 mm. During dynamic mechanical analysis, the sample was kept inside a chamber with water-soaked cotton to minimize water evaporation. Data were collected under controlled stress (30τ) and frequency of 3.0 rads^-1^.

### Fourier-transform infrared (FTIR) spectroscopy

The FTIR spectra of hydrogel solutions (3.0 wt% of mPA, M10/mPA, and H5/mPA in deionized water) were obtained using a FTS-800 system (Thermo Fisher, USA) equipped with attenuated total reflectance (ATR). The samples were balanced for 20 min at 37°C and spectra was record.

### Transmission electron microscopy (TEM)

The nanoscale morphology of MNP and HAP was examined by TEM. For TEM analysis, an instrument (H7100, Hitachi, Japan) operating at 120 keV was used. For sample preparation, a drop of a diluted dispersion of MNP or HAP was placed on a Formvar coated copper grid and left to dry on a filter paper. Before sample analysis, the copper grid was put into the drying oven to ensure low moisture.

### Scanning electron microscopy (SEM)

The pore structure and surface morphology of scaffolds were observed by SEM. Micrograph images of gels at 37°C were taken after polymer aqueous solutions (1 mL, 3.0wt %) were transferred from 25°C to 37°C and stored for 20 min in the oven. The gels were then quenched with liquid nitrogen at –196°C and freeze-dried. SEM images were obtained by a field emission scanning electron microscopy instrument (JSM-6700F, JEOL Ltd., Japan).

### Laser ablation inductively coupled mass spectrometry (LA-ICPMS)

The dispersion characteristic of inorganic nanoparticles in hydrogels was analyzed by LA-ICPMS. Briefly, 100 μL of the gel solution was transferred to a cover glass. The size and shape of the test samples were similar to those formed using a 48 well plate. Then, the slide was placed into the incubator to allow for gelation. A laser ablation system (Model: UP-213, New Wave Re- search Inc., Fremont, CA, Nd:YAG 213 nm wavelength) was used for sample ablation and the ablated fouling samples were introduced into a inductively coupled plasma mass spectrometer (7500a ICP-MS, Agilent Technology, Inc. USA) for element determination. The line scan mode was conducted under the following condition: a pulse rate of 10 Hz, a 150 μm diameter beam at a traveling velocity 100 μm/s, energy output 4 J/cm2, focused spot size 110 μm, transport Ar gas flow 1.01 L/min, dwell time 8 s, and intersite pause 1 s. The LA-ICP-MS scan range was 3000 μm × 2850 μm and a total of 900 lines covered the entire area of the tested gel sample. The raw data obtained from LA-ICP-MS were converted into a two-dimensional (2D) image by using the MATrix LABoratory (MATLAB) software.

### Cell culture

MC3T3-E1 pre-osteoblasts were cultured α-MEM supplemented with 10% FBS and 1% antibiotic/antimycotic. MC3T3-E1 pre-osteoblasts within passage 10 were used in this study to ensure pre-osteoblastic characteristics. Cells were grown in a humidified atmosphere of 5% CO_2_ at 37°C. The osteogenic media consisted of α-MEM supplemented with 10 mM β-glycerol phosphate, 1*10^−7^ M dexamethasone, and 50 μg/mL L-ascorbic acid. The culture medium was changed every 3 day till the cell density reached 80–90% confluences. The supernatant was then removed and cells washed with 10 mL of PBS three times to remove residuals from the medium, followed by treatment with 0.25% trypsin-EDTA solution. A cell pellet was collected after centrifugation at 750 rpm for 10 min, with supernatant discarded. A cell suspension was made by mixing the cell pellet with 1 mL cell culture medium and re-suspended in three 100 mm tissue culture plates.

### Gels encapsulated cells and *in vitro* culture

For the *in-vitro* hydrogels encapsulated-cells studies, mPA polymer was UV sterilized over night. mPA polymer was dissolved in deionized water and mixed with different inorganic nanoparticles as described above. After 3 days of culture, the cells were passaged and directly encapsulated to the mPA, H5/mPA, and M10/mPA polymer solutions at a final concentration of 5*10^3^ cells/well. The solutions were manually mixed, and allowed to gel at 37°C in an incubator for 30 min before *in vitro* culture. The cell-containing hydrogels and controls were placed in 0.5 mL medium in 48-well tissue culture plates and cultured for 7, 14, and 21 days. Medium was changed every 3 days by removing 250 μL medium and replenishing 250 μL fresh medium. At the desired time points, samples were processed for Live/Dead staining and real time RT-PCR assay (n = 3).

### LIVE/DEAD assay

Cell viability in the HAP or MNP co-culture system with or without gel encapsulation was determined using the LIVE/DEAD kit, respectively. At the desired time points, samples from each system were followed by removal of the medium and PBS washing for three times. The assay reagent was prepared by mixing 2 μL ethidium homodimer-1 (4*10^-6^M) and 1 μL calcein acetoxy methyl ester (2*10^−6^ M) in 1mL PBS. Added 200 μl reagent to each well in 48-well plates for staining live (green) and dead (red) cells. The whole process was performed in dark and incubated with the reagent at 37°C for 30 min. After the incubation period, the stained cells were viewed under a fluorescent microscope. The excitation/emission wavelength were 528/617 nm for ethidium homodimer-1 and 494/517 nm for calcein acetoxy methyl ester.

### Static magnetic field (SMF) exposure system

SMF-exposed cells and control cells were first placed in an incubator (NUAIRE, USA). Neodymium (Nd_2_Fe_14_B) magnets with a diameter of 8 cm and a thickness of 1 cm were used to produce the SMF. The average magnetic strength density on the surface was monitored by a handheld Gauss meter (EFA 3; B-field sensor, Wandel & Goltermann, Germany) and was shown to have an average strength of 0.4 T (range: 0.38–0.43 T). The density of SMF strength is in the range suitable for clinical test. For all exposure experiments, cultured plates were placed directly on the surface of the permanent magnets. Control cells were placed in the same incubator, but in an area with magnetic strength similar to the natural environment. To probe the effect of a moving magnetic field, we established a homemade moving magnetic system. The same magnets were used as for the SMF test, but the cell culture plates were placed on an orbital shaker inside the incubator. The daily magnetic exposure time and plate shake rate were 1 hour and 100 rpm, respectively [34].

### Cell proliferation and differentiation assay

For quantitative real-time polymerase chain reaction (PCR), the cell and cell-hydrogel were added TRIsure reagent according to protocol to extract total RNA at predetermined time points. Then, an all-in-one cDNA synthesis supermix kit was used for reverse transcription of total RNA to cDNA, which was stored at -20°C prior to use. Three different genes were analyzed including alkaline phosphatase (ALP), osteocalcin (OCN), and osteopontin (OPN) using a real-time PCR system (ABI Prism 7300, Thermo Fisher, USA). Glyceraldehyde-3-phosphate dehydrogenase (GAPDH) was selected as the housekeeping gene. Table 1 lists the primer sequences used.

**Table 1:** Primer pair sequences used in real-time RT-PCR [35].

Genes	Oliginucleotides sequence (5’- 3’)
Alkaline phosphatase (ALP)	F: GTTGCCAAGCTGGGAAGAACAC
	R: CCCACCCCGCTATTCCAAAC
Osteocalcin (OCN)	F: AGGGAGGATCAAGTCCCG
	R: GAACAGACTCCGGCGCTA
Osteopontin (OPN)	F: GACCACATGGACGACGATG
	R: TGGAACTTGCTTGACTATCGA
GAPDH	F: CCCTGTTGCTGTAGCCGTA
	R: CCGGTGCTGAGTATGTCG

### Statistical analysis

At least three replicates were performed for each test condition. The results were presented with error bars as the mean +/- SD. Statistical analysis was performed using the Student's t-test, p values < 0.05 were considered as statistically significant.

## Results and discussions

### Synthesis of di-block copolymer mPA and characterization

**Fig 1:**
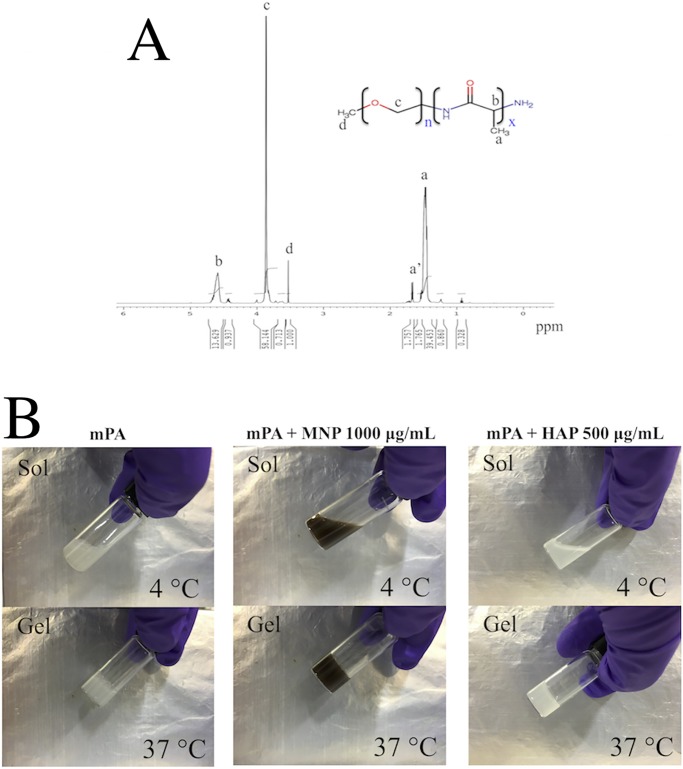
(A)^1^H-NMR spectrum of mPA (CF_3_COOD). (B) Photographs of sol (4°C) and gel (37°C) states of the injectable hydrogel solution.

The ^1^H-NMR spectrum of mPA is shown in Fig 1A. Five characteristic peaks in the spectrum were used to calculate the molecular weight (Mn) and composition of the polymer. In the NMR spectra, the methyl protons (CH-CH_3_) and methine protons (CO-CH-CH_3_-NH_2_) of poly(alanine) at 1.4 to 1.6 ppm and 4.5 to 4.7 ppm, respectively, were correctly found to be at a ratio of 3:1. The terminal methyl group at 1.6 to 1.7 ppm has a slightly downfield shift due to its close proximity with amine. Methylene protons (O-CH_2_-CH_2_) and the terminal methyl protons (CH_3_-O) of mPEG were found at 3.8 to 4.0 ppm and 3.5 ppm, respectively.

Mn and Mw of the copolymer were determined by GPC and ^1^H-NMR respectively. Using the integration ratio between methylene protons on mPEG and methyl protons on poly(alanine) Mw can be calculated, which was approximately 4500 Da.

The GPC of the mPA showed homogenous composition of the molecular weights with a polydispersity index (Mw/Mn) of 1.09, and the number average molecular weight (Mn) and weight-average molecular weight (Mw) determined with PEG standards are 2275 and 2490, respectively.

Finally, we selected 3.0 wt % injectable hydrogel solution in current study. The photographs of the sol (4°C) and gel (37°C) states prepared from the injectable hydrogel solution (3.0 wt %) are shown in Fig 1B.

### Characterization of inorganic nanoparticles

**Fig 2:**
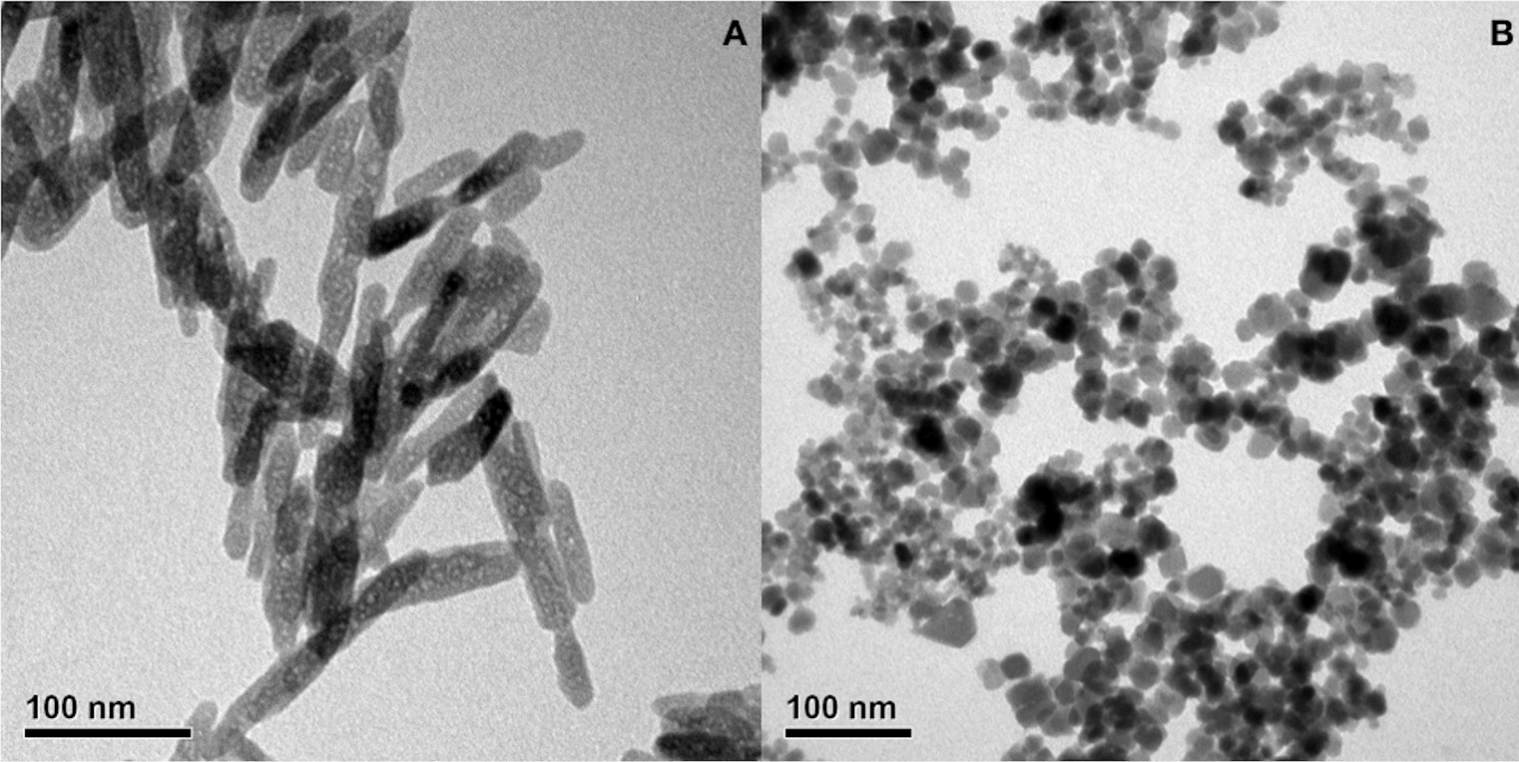
TEM images of (A) MNP and (B) HAP; the diameter MNP was approximately 10 nm and the size of HAP was approximately 10 nm in width and 100–150 nm in length.

TEM micrographs of HAP and MNP nanoparticles are shown in Fig 2. As revealed, the microstructure of HAP nanoparticles was needle-like with a mean crystallite size of 10 nm in diameter and 100–150 nm in length. The morphology of MNP nanoparticles was spherical with a mean particle diameter of approximately 10 nm.

The stability of nanoparticles in suspension is controlled by hydrophobicity- hydrophilicity, and van der Waals forces. Most biomedical applications require chemically stable and uniformly sized nanoparticles that are well dispersed in water. In general, nanometer size particles aggregate in suspension due to van der Waals forces in order to reduce the total surface or interfacial energy. Consequently, such aggregation can hamper the efficacy of nanoparticles in many applications such as drug delivery, catalysis, and detection due to low surface area and large sizes [36]. Surface modification of nanoparticles can be carried out either during their synthesis or in a post-synthesis process. Typically, polymers on the particle surface ensure particle stability and improve its dispersibility [37, 38]. These polymer coatings are generally required to be biocompatible and hydrophilic. In this study, dispersive MNP was prepared by reacting iron (II) and iron (III) in an ammonia solution with the addition of TMAH to chemically stabilize the MNP in a colloidal solution. For comparison, HAP was synthesized without the addition of TMAH.

### The dispersion of inorganic nanoparticles in the hydrogel

**Fig 3:**
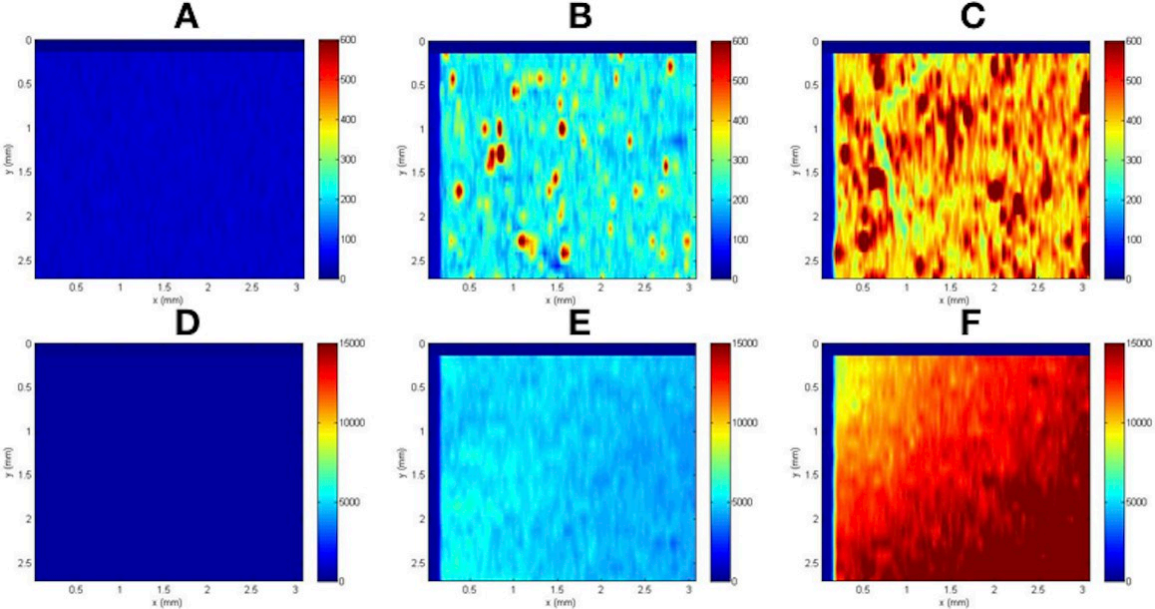
Elemental mapping of inorganic nanoparticles in the hydrogel. (A and D: mPA, B: H5/mPA, C: H10/mPA, E: M5/mPA, F: M10/mPA) Color bar on the right side indicates element intensity, with blue and red corresponding to low and high concentration respectively.

LA-ICP-MS is a powerful and direct solid-sampling analytical technique that allows for highly sensitive and precise elemental and isotopic analysis of materials [39, 40]. In this study, LA-ICP-MS was adopted to observe the elemental distribution of encapsulated inorganic nanoparticles in the hydrogels. Fig 3 shows the spatial distribution of studied elements in mPA, M10/mPA and H5/mPA hydrogels. As observed, elemental distribution became inhomogeneous when nanoparticle concentration was increased beyond 500 μg/mL. On the other hand, MNP were better distributed than HA at high concentration. In the H5/mPA group, the intensity at most areas was 100 to 200. However, specific areas had an intensity difference of approximately 2 to 5 times, indicating aggregation in local areas. In a comparison, the intensity of M5/mPA was homogenous at a low concentration. The intensity difference of M10/mPA was approximately 1.5 times, which is lower than those of of H5/mPA and H10/mPA. These data suggest that HAP tends to aggregate in the hydrogel due to its hydrophobicity.

### Cell toxicity of inorganic nanoparticles

**Fig 4:**
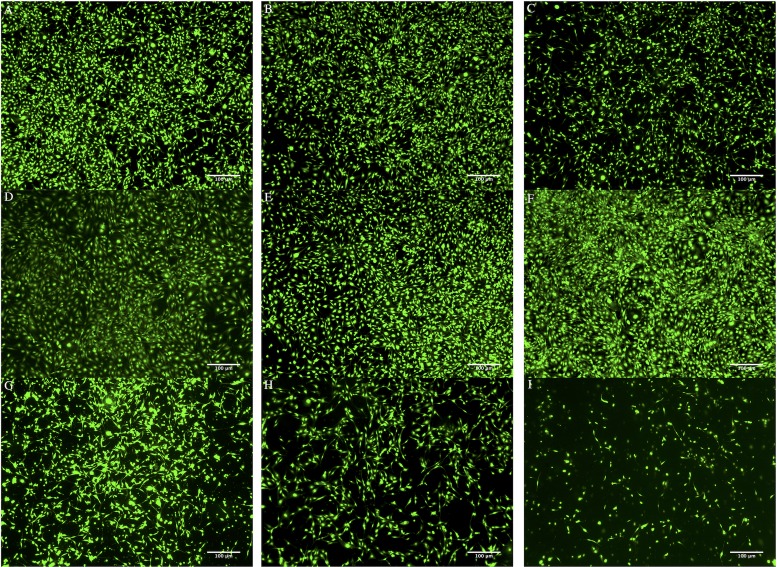
After day 7, the cell viability of co-culturing with different inorganic nanoparticles: (A) control, (B) MNP 100 μg/mL, (C) HAP 100 μg/mL, (D) MNP 500 μg/mL, (E) MNP 750 μg/mL, (F) MNP 1000 μg/mL, (G) HAP 500 μg/mL, (H) HAP 750 μg/mL, and (I) HAP 1000 μg/mL.

Cell proliferation was studied after 7 days of co-culture with different concentrations of inorganic nanoparticles. Fig 4 shows the LIVE/DEAD staining of MC3T3-E1 co-cultured with different concentrations of HAP and MNP.

According to all these figures, it is observed that (1) the cell viability of MNP or HAP with low (100 μg/mL) concentration is high, (2) for MNP, viability increased with concentrations (Fig 4D–4F), so 1000 μg/mL was the highest one, (3) for HAP, viability is highest in the 500 μg/mL group (Fig 4G–4I).

This result contradicts with previous studies where researchers demonstrated the positive effect of HAP on osteoblast proliferation and differentiation [41–44]. However, other researchers provided evidence that cell viability is related to HAP concentration. The HAP induced a negative effect on osteoblast functions which strongly suggests phagocytosis process. The phagocytosis process produces an accumulation of calcium in mitochondria and eventually leads to mitochondria lysis and cell death [45]. Therefore, selecting the better concentration range of HAP is crucial for improving osteoblast activity without compromising cell viability. Finally, concentrations of HAP and MNP were fixed at 500 μg/mL and 1000 μg/mL, respectively.

### Hydrogel microstructure

**Fig 5:**
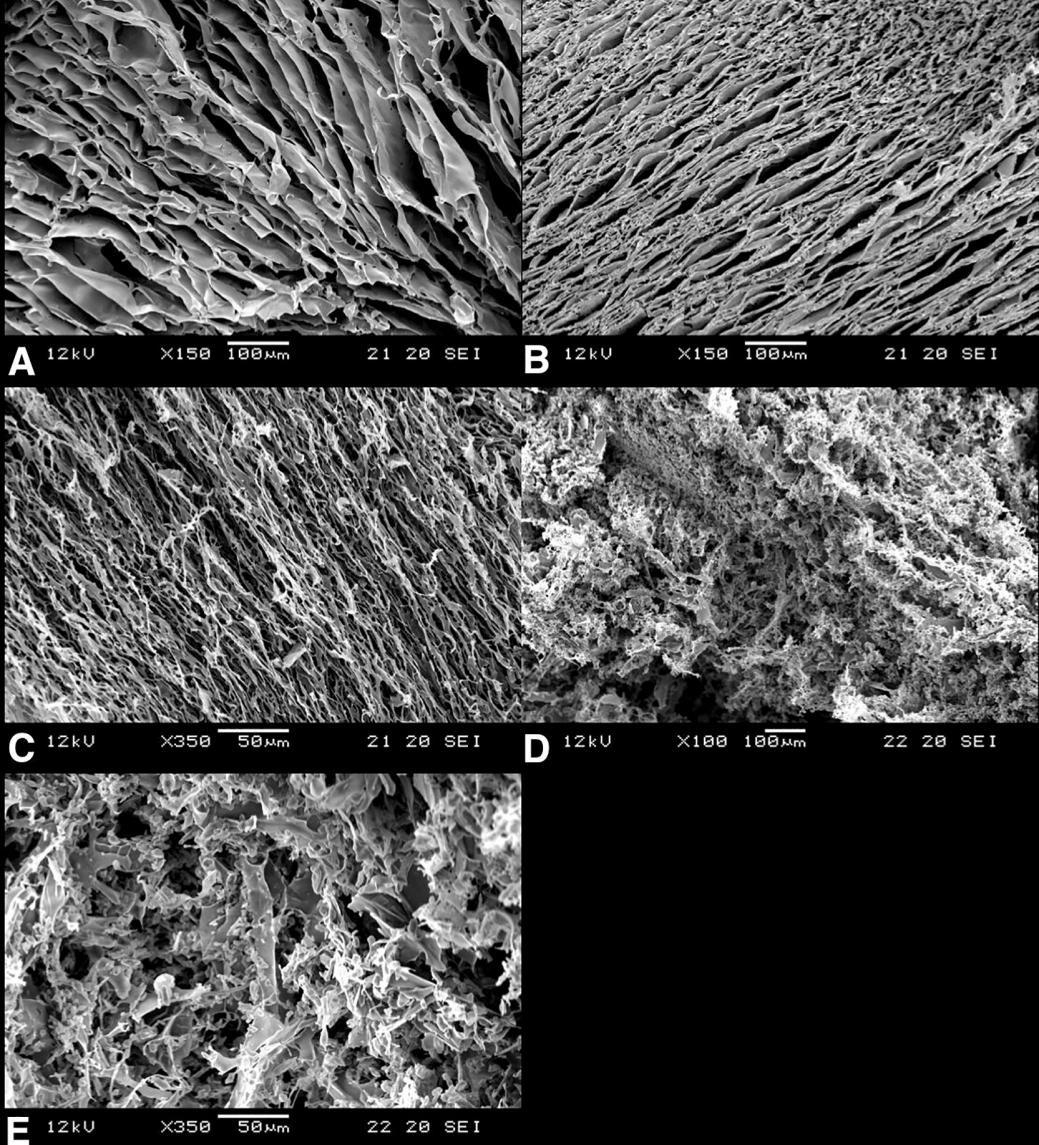
The SEM images of (A) mPA (blank), (B) M10/mPA (mPA+ 1000 μg/mL MNP), (C) H1/mPA (mPA+ 100 μg/mL HAP), (D) H5/mPA (mPA + 500 μg/mL HAP) hydrogels, and (E) the magnification of H5/mPA.

To examine the surface and interior morphologies of the hydrogels, SEM microscopy was adopted. Fig 5A, 5B, 5C and 5D show the microstructure of lyophilized 3% hydrogels of mPA, M10/mPA, H1/mPA, and H5/mPA respectively. As shown in Fig 5A, 5B and 5C, lyophilized hydrogels exhibited a plate-like 3D structure. However, when a high concentration of HAP was used, the plate-like structure became more porous. The porous structure allows not only the transport of nutrients, oxygen, carbon dioxide, and metabolite but also provides space for cell proliferation during 3D cell culture [46]. The pore size differed depending on the type of encapsulated inorganic nanoparticles [11, 47]. When particles encapsulated in di-block hydrogels are confined between two surfaces, the effects of chain stretching play an important role. *Lee et al*. [48] showed that a symmetric AB diblock copolymer confined between two surfaces forms a lamellar structure. The micro domain of stripes are oriented either parallel or perpendicular to the layers. *Lin et al*. [49] experimental studies showed precisely this behavior of mediated interfacial interactions, and reoriented micro domains. Finally, nanoparticles were excluded to the surfaces and caused the micro domains to orient normal to the surface. In our study, we observed a similar behavior when different concentrations of nanoparticle were encapsulated into the hydrogel. As shown in Fig 5C, low concentration of HAP did not change the hydrogel structure. However, a porous transformation was noted in Fig 5D and 5E. This suggests that high concentration of HAP prevents adequate dispersion and thus HAP were excluded to the hydrogel surface. The aggregation of HAP likely destroys the sheet-like structure, causing the gel to adopt a more porous structure.

Although the incorporation of HAP increased the porosity of the scaffold, high concentration HAP decreased the cell viability. Therefore, proper concentrations of MNP and HAP were chosen, as mentioned before.

### Sol-gel transition and FTIR measurement for secondary structure

**Fig 6:**
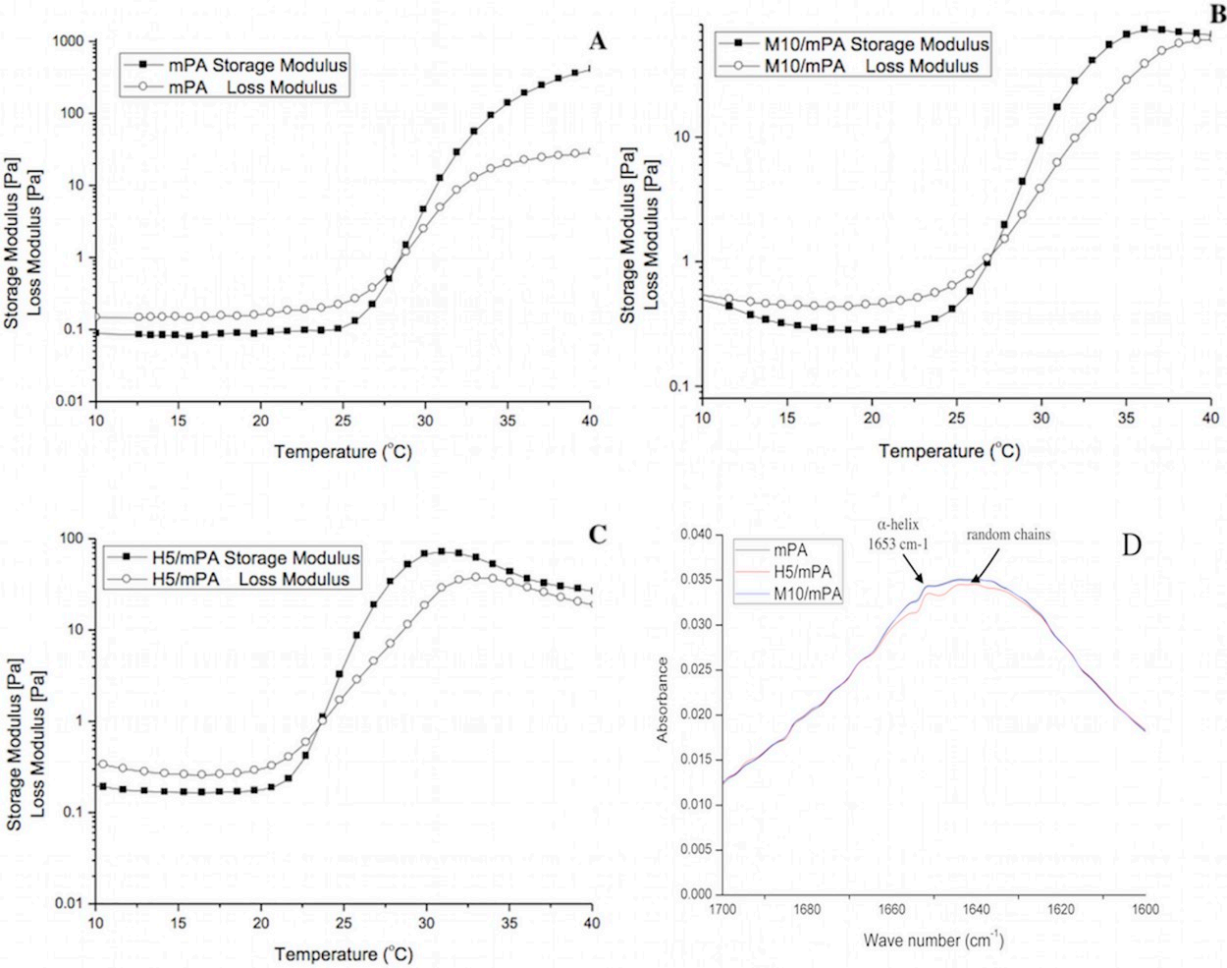
Dynamic rheology measurements: Storage modulus (G′) and loss modulus (G′′) of (A) mPA, (B) M10/mPA, (C) H5/mPA as a function of temperature. (D) The FTIR spectra of mPA, H5/mPA, and M10/mPA hydrogels.

Rheological properties of a 3% (w/v) aqueous solution of various composite hydrogels are as shown in Fig 6A, 6B and 6C. The gelation temperature corresponding to the crossover point of G’ and G” were at 28.0°C, 27.0°C, and 23.5°C respectively for mPA, M10/mPA, and H5/mPA. *Su et al*. [50] studied the gelation and bioactivity behavior of an injectable silk fibroin hydrogel with laponite nanoplatelets (LAP), concluding that LAP served as both a medium to accelerate the hydrophobic interactions among silk fibroin and a disruptor to limit the growth of *β*-sheet domain during the gelation. Corresponding secondary structures found in nanocomposite hydrogels at 37°C are shown in Fig 6D. Result shows that HAP incorporation was disruptive and decreased the proportion of random coils and *α*-helix. The lowering of storage modulus, as shown in Fig 6A, 6B and 6C, could be attributed to the same mechanism.

### LIVE/DEAD staining of gel-encapsulated cells

**Fig 7:**
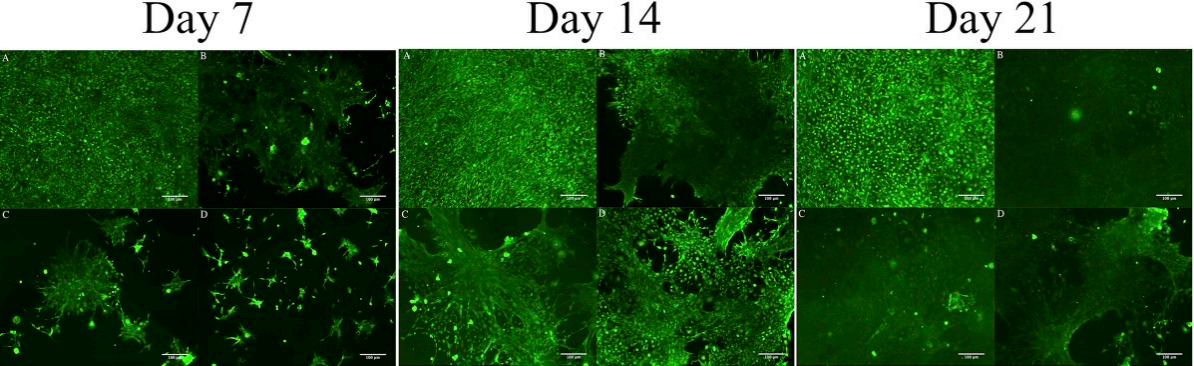
The live/dead staining images of (A) control (2D culture), (B) mPA, (C) H5/mPA, and (D) M10/mPA after day 7, 14, and 21 cultured. (B, C, D were 3D culture).

LIVE/DEAD staining revealed that the cells were metabolically active and homogenously distributed within the hydrogel matrix at different time point. (Fig 7) With extended culture, the number of viable cells increased in all hydrogels. However, in 3D culture groups (Fig 7B, 7C and 7D), osteoblasts migrated into clusters on day 21 and the hydrogel became opaque compared to the control (Fig 7A), likely owing to the accumulation of ECM material and/or calcium [51]. The ECM is a combination of numerous components, which jointly provide a wide range of functionality. Except for providing structural support, the ECM is also a channel for cell-cell communication [52], and a driving force behind organogenesis [53].

Previous studies also demonstrated that ECM mineralization could be induced by the addition of inorganic nanoparticles that provide attachment sites to enhance integration with surrounding bone tissue [54–56]. Furthermore, we also observed the characteristic of MC3T3-E1 cells differentiation such as microfilaments. The hydrogel encapsulated with HAP (Fig 7C in day 14) and MNP (Fig 7D in day 14) compared with mPA (Fig 7B in day 14), we observed more microfilaments in groups with nanoparticles than mPA group. This kind of change in cell morphology was also observed in previous studies [57–59].

### Biochemical analysis and real time-PCR of gel-encapsulated cells under magnetic field

#### Static magnetic field

**Fig 8:**
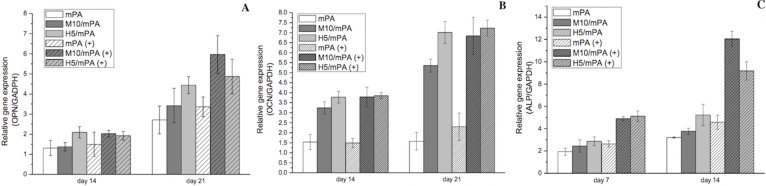
(A) OPN, (B) OCN, and (C) ALP gene expressions as a function of time. GAPDH was used as the housekeeping gene. “+” denotes the presence of a static magnetic field.

The three main characteristics of cellular growth during differentiation from pre-osteoblasts to osteoblasts are cell proliferation, ECM maturation and mineralization [60]. The expression of osteoblast proliferation and differentiation markers, OPN, OCN and ALP, were analyzed by real time PCR as shown in *Fig 8*. ALP activity is an early marker (before 14 days) for osteogenic differentiation and regulates phosphate metabolism [61]. In our experimental design, the LA-ICPMS test samples were used the 48 well plate space to analysis the dispersion of nanoparticles in the hydrogel. So, we also used the 48 well plate to form the similar hydrogel to conduct our gene expression experiments. As shown in Fig 8, ALP activity was induced after 14 days of culture in all test groups. A significant 1-fold increase in ALP activity was observed when a static magnetic field was applied on M10/mPA. Injectable polypeptide hydrogels have been shown to provide a suitable environment for cell attachment and growth by mimicking the structure of ECM [8, 62–65]. In this study, MNP was incorporated into the polypeptide hydrogel, to render responsively to an applied magnetic field. MNP as an intrinsic component of the polypeptide hydrogel allows the introduction of magnetic force inside the hydrogel when an external magnetic is introduced. This magnetic force continuously stimulates proliferation of osteoblasts and secretion of new ECM [23]. Previous studies reported that in common cell culture systems, weak magnetic force with an intensity of 0.1–10 T accelerated osteoblast differentiation [29, 66, 67].

After the expression of ALP, osteoblasts begin to develop and mature. At the stage of bone matrix maturation and mineralization, osteoblasts express high levels of OPN which modulates the mineralization of the bone matrix. OPN is a highly acidic phosphoprotein that is related to cell adhesion [68]. Meanwhile, because OPN is a secreted protein, high levels of ECM easily sequester OPN within the polypeptide hydrogel. Results show that without an applied magnetic field, cells cultured in H5/mPA showed higher expression level of OPN beginning on day 14 and continued to day 21 compared to the other groups. In addition, OPN upregulation was observed with prolonged culture. The expression of OPN in all groups was increased approximately two- to three-folds.

However, when a static magnetic field was applied, cells cultured in M10/mPA exhibited higher expression level of OPN compared to H5/mPA and the other groups. OCN is a small protein produced by osteoblasts during the development period of extracellular matrix mineralization and it is also a late biomarker. As shown, its expression was highest after 21 days of incubation. A two-fold increase was noted in M10/mPA and H5/mPA from day 14 to day 21. Although the precise function of OPN and OCN are not fully elucidated, previous studies demonstrated that they play an important role in calcium decomposition and mineralization [69].

The gene expression of ALP, OPN, and OCN in the H5/mPA group was higher than that of other groups and this can be attributed to the positive effect of calcium ions released into the culture medium by HAP. On the other hand, the application of external magnetic fields shown to positively affect bone regeneration in all group, though not as large as those HAP or MNP induced. The major effect of SMF on osteoblast differentiation is inducing membrane reorientation and distortion, which alter the diamagnetic isotropic properties [29]. The SMF changes cell-cell contact properties in confluent or post-confluent cultures. This change correlates to the reorientation of phospholipids which results in the deformation of the lipid bilayer or proteins embedded on the membrane [70, 71].

#### Moving magnetic field

**Fig 9:**
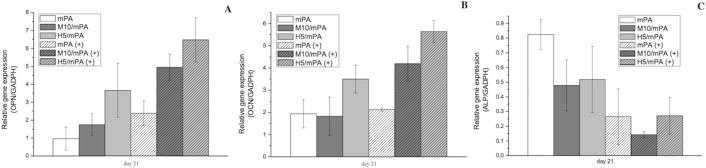
Real time RT-PCR analysis of (A) OPN, (B) OCN, and (C) ALP gene expressions on day 21 with the application of a moving magnetic field. GAPDH was used as the housekeeping gene. The representation “+” means applied the moving magnetic field.

A moving magnetic field was used to stimulate the culture osteoblasts and a pulsed exposure scheme was designed to prevent overheating of the mechanical shaker. By referencing previous studies, a daily exposure time of 60 minutes for 21 days was selected [72, 73]. Compare to the results of static magnetic field, similar findings were observed with the use of a moving magnetic field, as shown in Fig 9. When a moving magnetic field was applied, OPN and OCN expressions were increased. Encapsulated MNP likely acted as substrates that attract growth factors in a magnetic guiding process [26]. Furthermore, encapsulated MNP also is capable of acting as a cross-linking agent, providing chemical-physical stabilization [74].

## Conclusions

In this work, a simple method for encapsulating different inorganic nanoparticles in a polypeptide hydrogel was reported. Cell culture results confirmed the compatibility of these hydrogels with pre-osteoblasts. PCR results demonstrated that HAP acted as a proliferation cue in H5/mPA hydrogel and ALP expression was upregulated in the same system. Application of a magnetic field to cells encapsulated in MNP-incorporated polypeptide hydrogel showed positive enhancement to osteoblast differentiation. The idea of nano-particle incorporation to cell-encasulating hydrogel was proved to be beneficial for bone regeneration in principle, and the magnetic field’s positive effects on osteoblast differentiation were demonstrated.
